# Early recognition of pulmonary complications of sickle cell disease

**DOI:** 10.15537/smj.2023.44.1.20220636

**Published:** 2023-01

**Authors:** Rayyan M. Almusally

**Affiliations:** *From the Department of Internal Medicine, King Fahd Hospital of the University, Imam Abdulrahman Bin Faisal University, Khobar, Kingdom of Saudi Arabia.*

**Keywords:** sickle cell, pulmonary hypertension, acute chest syndrome, obstructive sleep apnea, pulmonary embolism, pneumoni

## Abstract

Sickle cell disease (SCD) is an inherited hematological disorder with multiple-organ involvement. The pulmonary complications of SCD are associated with significant morbidity and mortality. This article presents an important review of acute and chronic pulmonary complications, including acute chest syndrome, pneumonia, pulmonary thromboembolism, pulmonary fat embolism, chronic sickle cell lung disease, and pulmonary hypertension, in patients with SCD. Bronchial asthma and obstructive sleep apnea in relation to SCD are discussed in this article. Early recognition of pulmonary complications leads to early therapeutic interventions and improvement of the overall treatment outcome.

**S**ickle cell disease (SCD) is an autosomal recessive hemoglobinopathy with multisystem involvement that affects approximately one in 600 African Americans.^1^ The prevalence of SCD in Saudi Arabia has reached 1.4% and 2-27% for carriers, depending on the geographic area; the highest prevalence is in the Eastern province.^2^ Sickle cell disease is named with the word “sickle” because of the characteristic sickle-shaped erythrocytes described by Herrick in 1910. Sickle hemoglobin (HbS) was identified through electrophoresis by Pauling in 1949. Sickle hemoglobin is a result of the substitution of valine for glutamic acid (GAG to GTG) at the sixth amino acid of the beta-globin chain in chromosome 11.^3^ This change leads to the formation of the HbS tetramer during the deoxygenation phase, which produces cellular rheological abnormalities that include rigid cells with distorted shapes and membrane damages, which could subsequently affect the microvascular blood flow, causing vaso-occlusion at the capillary level and hemolysis. This polymerization increases with higher intracellular concentrations of HbS and decreases with elevated fetal hemoglobin (HbF) levels.^1^

Sickle cell disease is associated with complications involving various systems, most often the respiratory system.^4^ The aim of this review article was to discuss the pathophysiology, clinical manifestations, and diagnosis of and treatment options for the pulmonary complications of SCD, such as acute chest syndrome, pneumonia, pulmonary thromboembolism, pulmonary fat embolism (PFE), sickle cell chronic lung disease (SCLD), and pulmonary hypertension (PH).^5^

## Definition

Acute chest syndrome (ACS) is a potentially severe acute pulmonary complication occurring in up to 20% of hospitalized patients, usually within the first 72 hours of admission for vaso-occlusive pain crisis (VOC).^6^ It is the most common cause of intensive care unit (ICU) admission among adults and children.^7,8^ It is defined as a new pulmonary infiltrate on chest radiography (CXR) that involves at least one complete lung segment and may be associated with pleuritic pain, fever (>38.5°C), cough, tachypnea, wheezing, and hypoxemia (PaO_2_<60 mm Hg).^8^

**Figure 1:**
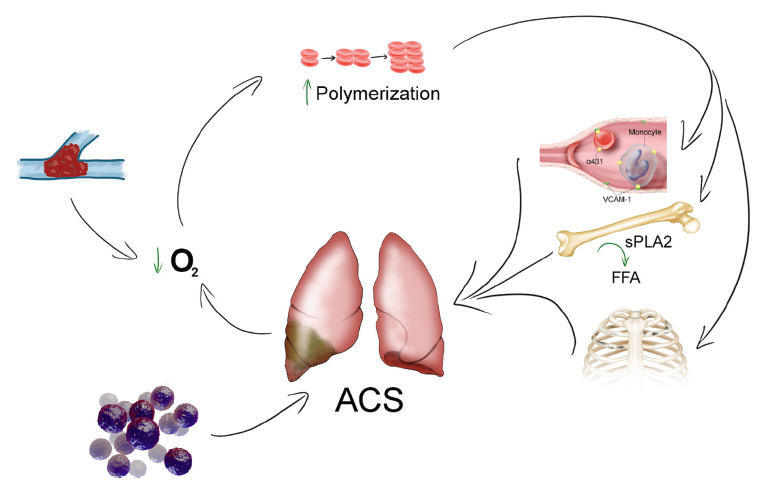
- The pathophysiology of acute chest syndrome. Sickle haemoglobin polymerization increased during dexoygenation in sickle cell disease patients and because of increased hemolysis rate, nitric oxide might be inactivated by free plasma hemoglobin and reactive oxygen that leads to microvascular occlusion by sickle cell as well as leukocytes mainly monocytes. Secretory phospholipase A2 converts bone marrow fats into free fatty acids which can accumulate in lung vasculature. Rib infarction due to vaso-occlusion could result in hypoventilation and atelectasis. Micro-organisms known cause of pneumonia that precipitate acute chest syndrome. Thromboembolism is an example for hypoxemia. Acute chest syndrome is the end result of the precipitating factors and hypoxemia which is directly worsen it as well.^1,9^ VCAM-1: vascular-cell adhesion molecule 1, sPLA2: secretory phospholipase A2, FFA: free fatty acids, ACS: acute chest syndrome.

Acute chest syndrome could occur secondary to pneumonia, thromboembolic phenomena, fat embolism, microvascular occlusion, or rib infarction.^1,9^ It is more common in children and may follow VOC with severe limb pain, chest pain, and fever. The risk of ACS doubles when the baseline Hb level is >12 g/dL, which indicates relatively hyperviscous blood. On the other hand, a sudden decrease in Hb to <0.78 g/dL with an increase in lactate dehydrogenase (LDH) level can lead to ACS, which could reflect the impact of hemolysis (Figure 1). The baseline platelet count in patients with SCD could be as high as >400×103/µL because of spleen dysfunction or surgical splenectomy. Decreased platelet count (<200×103/µL) is a predictor of ACS, which could be linked to platelet consumption in microvascular occlusion.^8^ However, an increase in HbF from 5% to 15% may reduce the incidence of ACS by approximately 50%.

A multicenter prospective study evaluated ACS etiologies. It studied 671 episodes of ACS in 538 patients, of whom 443 had only one episode; 69, 2 episodes; 16, 3 episodes; 8, 4 episodes; and 2, 5 episodes. The etiology could not be identified in nearly half (45.7%) of the episodes. The known causes were infarction (16.1%), isolated fat embolism (6%), and infectious pathogens (29.4%). An overlap between fat embolism and an infectious agent was observed in 2.8% of the cases. The most frequently isolated pathogens were *Chlamydia pneumonia* in 71 episodes, *Mycoplasma pneumoniae* in 51 episodes, and respiratory syncytial virus in 26 episodes.^8^ Given that these data were belonged to the United States of Amercia’s population in the 1990s, management decisions should depend on the local and current available microbiome data.

## Clinical manifestations

Acute chest syndrome could present clinically with chest pain, dyspnea, cough, hemoptysis, wheezing, and fever (Table 1).^10^

**Table 1:** - Clinical manifestations and some investigations for patients with acute chest syndrome.

Variables	Precentages of ACS episodes
***Symptoms***	
Fever	80%
Chest pain	76%
VOC	74%
Cough	70%
Shortness of breath	38%
Productive cough	24%
Chills	18%
Wheezing	11%
Hemoptysis	2%
***Vital signs***	
Temperature >38°C	36%
Respirations >30/minute	36%
Pulse >140 beats/minute	15%
***Examination***	
Rales	57%
Normal exam	35%
Dullness to percussion	31%
Rhonchi	16%
***Chest X-ray findings***	
Infiltrates	94%
Effusion	50%
***Arterial blood gas***	
PaO_2_ <80 mmHg	88%
PaCO_2_ >40 mmHg	46%

Data are collected from 1708 ACS events.8,10,11,19,53 ACS: acute chest syndrome, VOC: vaso-occlusive pain crisis, PaO_2_: partial oxygen pressure, PaCO_2_: partial pressure of carbon dioxide

## Investigations

**Figure 2:**
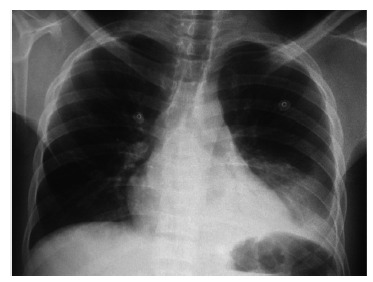
- Chest X-ray demonstrate left lower lobe opacification with minimal pleural effusion in a 16 years sickle cell disease patient presented with acute chest syndrome. The patient improved markedly after exchange blood transfusion.

The characteristic laboratory findings of ACS include a decrease in Hb level by approximately 0.7 g/dL from baseline and an increase in white blood cell count by 69%. The platelet count decreases to <200×103/µL. In arterial blood gas (ABG), PaO2 reaches <70 mm Hg but might be reduced to as low as <60 mm Hg in 20% of patients. However, PaCO2 usually remains within normal limits. A microbiology workup could reveal *Streptococcus pneumoniae* and *Hemophilus influenzae*. *Staphylococcus aureus*, *Salmonella*, *Enterobacter*, and *Clostridia* are rarely isolated.^8^ In radiological studies such as chest radiography, infiltrates can be found most frequently in the lower lobes, which could be related to an atelectatic process. The infiltrates in approximately 33% of cases are bilateral. At admission, up to 35% of patients might have pleural effusion, which requires ruling out any infections (Figure 2). However, a normal chest radiography result does not necessarily exclude ACS. Therefore, a chest computed tomography (CT) scan may play an important role in certain circumstances. Contrast-enhanced CT can be ordered to rule out pulmonary embolism (PE) if it is suspected clinically.^8^ In febrile patients with SCD, chest radiography should be carried out to identify evidence of ACS even in the absence of clinical signs and symptoms of respiratory distress because up to one-third of patients might have normal lung examination results. Many physicians underestimate the incidence of ACS, which carry morbidity and mortality risks, in febrile patients with SCD.^11^

## Treatment

If ACS is suspected, therapy must be started as early as possible even in mild cases because the patient’s general health condition can rapidly deteriorate. The standard therapy for ACS includes supportive measures such as pain control, hydration, antibiotics, bronchodilators, supplemental oxygen, incentive spirometer, and blood transfusion. Parenteral opioids such as morphine are used for pain control, but high doses should be avoided because of the risk of respiratory depression and pulmonary edema.^12^ Fluid infusion 1.2 times the maintenance of one-half normal saline is recommended for the first 2 days to correct the hypovolemia. Patient monitoring is needed to avoid fluid overload and pulmonary edema.^10^ Owing to hypoxemia, the oxygen saturation (SaO_2_) of patients with ACS should be monitored continuously. Oxygen is required when the SaO_2_ decreases to <92% or partial oxygen pressure (PaO_2_) decreases to <70 mm Hg. Depending on the severity of the disease, patients may require non-invasive or invasive mechanical ventilation. Antibiotic use to prevent superimposed pneumonia should be considered.^8^ As the use of bronchodilators has been found to improve ACS in one-fifth of non-asthmatic patients, the concern that airway inflammation contributes to the bronchoconstriction in patients with ACS has increased.^8^ The definitive therapy is blood transfusion, which includes the transfusion of packed red blood cells (RBCs) or RBC exchange to replace the patient’s blood. In ACS attack, patients can be managed with simple transfusion by adding 2-4 units of packed RBCs within the first day, which would improve oxygenation (such as transfusion improved the SaO_2_ from approximately 86-93%). The Hb level should be maintained within ≤10 g/dL to prevent relative hyperviscosity. Exchange transfusion is required to reduce blood viscosity, vaso-occlusive crises, and hemolytic crises and to increase the oxygen-carrying capacity. It is administered for severe or rapidly deteriorating conditions to keep the HbS level at <30%, the Hb level at <10 g/dL, and the hematocrit level at approximately 30%. Nitric oxide (NO) has been used to manage ACS but does not show beneficial effects on the duration of the vaso-occlusive and hemolytic crises and the rate of ACS attacks.^13^

## Prevention

Incentive spirometry is an important intervention to prevent atelectasis and hypoxemia, which eventually decreases the incidence rate of ACS significantly. The education and observation of patients during the intervention improve compliance.^13,14^ The administration of hydroxyurea markedly reduces the recurrence rate of ACS, which makes it of a paramount importance.^15^ The usefulness of prophylactic transfusion in preoperative patients has been studied, and those who received blood transfusion had 3 ACS episodes, whereas those who did not receive prophylactic transfusion had 27 ACS episodes, indicating a significant risk reduction.^16^ Immunization against *Streptococcus pneumoniae*, *Neisseria meningitidis*, *Hemophilus influenzae* type B, and the influenza virus is of great value to reduce the risk of infections and ACS.^17,18^

## Pneumonia

The risk of pneumonia might be higher in patients with SCD given their tendency to develop infections after undergoing splenectomy for spleen complications or spleen dysfunction secondary to repeated VOC. This increases the possibility of infections with encapsulated organisms because of the opsonophagocytic dysfunction of the spleen caused by complement pathway disturbance.^19,20^ Organisms such as *Hemophilus influenzae*, *Streptococcus pneumonia*, and atypical organisms have been frequently isolated.^17^ The choice of an antibiotic regimen that includes third-generation cephalosporin and macrolide or fluoroquinolone is recommended depending on the regional guidelines.^18^ Viral infections should be suspected especially in the presence of bilateral infiltrates. Differentiation between pneumonia and ACS is challenging, and in case of a diagnostic dilemma, preventive management for both possibilities by simultaneously administering antibiotics and blood transfusion is recommended.^17^

## Pulmonary fat embolism

Microvascular sickling in the intramedullary cavity of the bone leads to fat necrosis and marrow embolism, which may penetrate the lungs as PFE. Pulmonary fat embolism is initially found in post-mortem biopsies from patients with SCD. This finding leads to further studies to search for its pathophysiological and clinical importance. In some post-mortem studies, the incidence of PFE is high, found in up to three-fourths of patients. Its clinical manifestations include respiratory distress, fever, delirium, confusion, and consciousness deterioration. As in non-SCD patients with PFE, half of SCD patients with PFE may have respiratory distress, severe hypoxemia, and neurological manifestations. Anemia and thrombocytopenia are also found. The level of secretory phospholipase A2 (an enzyme that converts phospholipids to free fatty acids) is high in such condition.^21^

Gurd’s criteria, which are used for long bone fractures, could be applied in diagnosing PFE.^21^ It requires one major and 2 minor criteria along with fat macroglobulinemia. The major criteria include respiratory distress, neurological manifestations, and petechial skin rash. The minor criteria are fever, tachycardia, jaundice, and retinal and renal involvements. Bronchoalveolar lavage may show fat droplets containing alveolar macrophages.^22^

## Treatment

The role of steroids in the prevention of PFE has been studied. Low doses of steroids, such as 9 mg/kg methylprednisolone, have reduced the incidence of PFE after long bone fracture. The mechanism of action of steroids against PFE associated with SCD is not well understood. Further studies are recommended to assess the effectiveness of corticosteroids in this complication.^23^ Exchange transfusion has been used, but no clear supporting evidence has been found regarding its effectiveness.

## Pulmonary thromboembolic phenomena

The prevalence of PE is extremely higher in patients with SCD than the general population aged <40 years.^24^ In autopsies, 22 cases of PE with and without infarction were found in the corpses of 72 patients who died of SCD.^25^ The mortality rate was 6%.^26^ pulmonary embolism can be the trigger of the onset of PH. Patients with PE may have dyspnea, pleuritic chest pain, hemoptysis, cough, associated calf pain, and edema.

Computed tomography pulmonary angiography is used to confirm the diagnosis. However, there is a concern that the use of ionic contrast might lead to the sickling process.^27^ To detect deep venous thrombosis, Doppler ultrasonography for the lower limbs and a ventilation-perfusion scan can be considered.^28^ Only after confirmation of the diagnosis can anticoagulation be initiated. The side effects of anticoagulation do not differ between SCD and other diseases. Given the risk of PE in hospitalized patients with SCD, prophylactic anticoagulation could be used in selected cases. Furthermore, thrombotic events were reported in patients with sickle cell traits.^29^

## Bronchial asthma and SCD

Bronchial asthma (BA) is an airway disease characterized by recurrent attacks of cough, shortness of breath, and wheezing. It affects up to 4% of adults in Saudi Arabia.^30^ The prevalence of BA in patients with SCD is similar to that in non-SCD patients, making it a coexisting disease rather than a sequela of SCD. The incidence rate of ACS is 4 times higher in asthmatic patients with SCD than in non-asthmatic patients.^31^ The relationship between BA and ACS is not well understood. Repeated episodes of ACS in non-asthmatic patients with SCD induce airway hyperresponsiveness that contributes to the wheezing heard on clinical examination. In a study that included 31 non-asthmatic adult patients with SCD, a methacholine provocation test revealed bronchial hyperreactivity in half of the patients.^32^

The relationship between BA and pain episodes is controversial. An cooperative study of sickle cell disease cohort study with more than 4000 patient-years showed a higher incidence rate of pain episodes among SCD patients with BA than among those without BA.^33^ By contrast, a retrospective cohort study carried out in France that included 297 children (1805 patient-years), of whom 25 had asthma, showed no difference in pain episodes between the patients with and without asthma.^34^ A study with 1963 patients, including children and adults (a total of 18,495 patient-years), with a follow-up period of around 10 years showed that BA reduced the survival of patients with SCD by almost 12 years compared with that of patients without BA (64.3 years vs 52.5 years).^35^ A study that included 39 patients with SCD showed a degree of bronchospasm during painful crises in admitted patients, which was associated with decreased peripheral eosinophilic count (mean cell count: 0.170 10^9^/L). This increased the suspicion of eosinophilic consumption in the pathogenesis of the disease.^36^

The management of asthma in patients with SCD is similar to that in the general population. Compliance to medications, avoidance of precipitating factors, and immunization against common respiratory pathogens are important to minimize complications.

## Sickle cell chronic lung disease

A chronic complication that has been classified into four stages (Table 2). The average age at diagnosis in the first stage is 23 years and last stage is 33 years. Half of patients die within 7 years of onset, and the causes of death are usually respiratory failure and myocardial ischemia. Risk factors include repeated episodes of ACS, aseptic bone necrosis in adult patients (age: >20 years), sickle cell painful crisis, chest pain, frequent hospitalizations, and low HbF levels. Arterial blood gas hypoxia, radiological changes suggestive of fibrosis, cor pulmonale electrocardiography changes, abnormal pulmonary function test (PFT) results, and PH as determined with right heart catheterization (RHC) are the diagnostic parameters of SCLD.^37^ Most patients have abnormal PFT results (up to 90%), which would show a restrictive pattern (74%) with a predicted total lung capacity of 55-85%. Obstructive patterns, mixed obstructive and restrictive patterns, and isolated low diffusing capacity for carbon monoxide have been reported.^38^ Unfortunately, most cases are diagnosed in the late stage. Early diagnosis leads to early therapeutic interventions and better treatment outcomes. Minimizing sickle cell crises and ACS and lowering the HbS concentration are preventive measures. Patients may require supportive therapies such as long-term home oxygen therapy and non-invasive mechanical ventilation.

**Table 2:** - Sickle cell chronic lung disease stages.

Stages	ABG	CXR	PFT	ECHO	PA pressure
1	NL	Increased interstitial marking	FEV1/FVC ratio <80%	NL	NL
2	NL	Fine interstitial fibrosis	FEV1/FVC ratio <60%	Balanced ventricular hypertrophy	NL
3	PaO_2_ <70 mmHg	Pulmonary fibrosis	FEV1/FVC ratio <40%	RVH, RA enlargement	Borderline
4	PaO_2_ <60 mmHg	Severe pulmonary fibrosis	Unable to do the test	Severe RVH, RAH	Elevated

Data are modified from Powars et al’s study.^37^ ABG: denotes arterial blood gas, CXR: chest X-ray, PFT: pulmonary function test, ECHO: echocardiogram, PA: pulmonary artery, NL: normal, FEV1: forced expiratory volume for the 1st second, FVC: forced vital capacity, PaO_2_: partial oxygen pressure, RVH: right ventricular hypertrophy, RA: right atrium, RAH: right atrial hypertrophy

## SCD and obstructive sleep apnea

A cohort study carried out with 53 pediatric patients with SCD (ages: 1.9-16.5 years) found that almost half of the patients had symptoms and signs suggestive of obstructive sleep apnea (OSA). Physiological studies revealed that 16% of the patients had abnormal hypoxemia (episodic or baseline hypoxemia). The study confirmed that nearly one-third of the patients had OSA. The cause of hypoxemia during sleep was hypoventilation secondary to adenotonsillar hypertrophy, which may be more common in patients with SCD than in healthy people. The mechanism of this phenomenon is thought to be the hypertrophy of the lymphoreticular system because of a non-functioning spleen.^39^

A previous study reported the case of a 12-year-old girl with SCD who had OSA based on her history, clinical examination result, and polysomnogram who reported frequent vaso-occlusive crises for the past 3 years. Tonsillectomy and adenoidectomy were carried out, subsequently improving the vaso-occlusive crises markedly, which in turn resolved her pain in around 2 years and cured her OSA.^40^

Sunil et al^41^ carried out a cohort study that included 32 adult patients with SCD whose Epworth sleepiness scale score was ≥10 and who underwent a full sleep evaluation. Of the patients, 44% had sleep-disordered breathing (SDB) documented by polysomnography, and more than 50% had insomnia and delayed sleep phase syndrome. The authors reported that 86% of the patients with SDB were using narcotics, which increased the concern regarding the adverse effects of the chronic use of narcotics.^41^

The risk factors of OSA in patients with SCD contributed mainly to tonsillar enlargement, adenoid hypertrophy, narcotic use, and cerebrovascular complications. Bone hypertrophy and thalassemic face changes may play roles in potentiating the upper airway obstruction in patients with coexisting thalassemia. Studies have included large numbers of SCD populations and are recommended to find evidence of a more significant association between SCD and OSA in order to establish a screening tool for patients with SCD.

## Pulmonary hypertension

It is considered a chronic complication of SCD. It has also been found in patients with acquired and hereditary hemolytic anemias (such as thalassemia, hereditary spherocytosis, and paroxysmal nocturnal hemoglobinuria). Pulmonary hypertension is defined as a mean pulmonary artery pressure of >20 mm Hg, a pulmonary capillary wedge pressure of <16 mm Hg, and a pulmonary vascular resistance (PVR) of >3 Wood units as measured using RHC. However, because of anemia, a PVR of >2 Wood units would be consider high in patients with SCD.^42,43^ On echocardiography, the peak tricuspid regurgitant jet velocity (TRV) is >2.5 m/second. Sickle cell disease patients with PH have lower mean pulmonary artery pressures than patients with idiopathic PH. Pulmonary hypertension in SCD has been moved from group 1 to group 5 of the World Health Organization classification because of its mixed pathophysiology.

The estimated prevalence of PH based on echocardiography results in adult patients with SCD reaches 30%, which would be approximately 9 million worldwide.^44^ These patients have higher mortality rates than other SCD populations. A previous study evaluated 141 patients, of whom 29% had PH, 7% had diastolic dysfunction, and 11% had both. The relative mortality risks were 5.1, 4.8, and 12, among these 3 groups of patients.^45^ The mortality rate increases by approximately 1.7-fold if the mean pulmonary artery pressure increases by 10 mm Hg.^46^

The pathophysiology of pulmonary arteriopathy is thought to be related to endothelial dysfunction because of nitric oxide dysregulation (secondary to cell-free plasma hemoglobin) and reactive oxygen species production, which can cause hemostatic activation and smooth muscle cell proliferation. In addition, because of chronic anemia, the cardiac output is high, increasing pulmonary blood flow. Risk factors include severe chronic hemolysis, advanced age, high blood pressure, presence of cardiac or renal disease, leg ulceration, priapism, and >10 blood transfusions.^44^

Patients with SCD-PH usually present with non-specific symptoms such as dyspnea on exertion, fatigue, lower extremity edema, or syncope. Dyspnea is sometimes interpreted falsely as a symptom of anemia.

**Figure 3:**
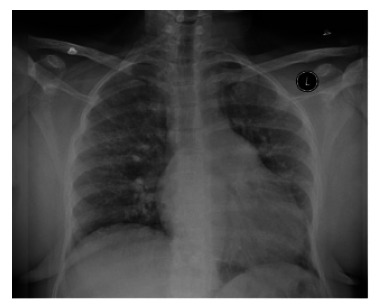
- Chest X-ray illustrates dilated main pulmonary artery and increased pulmonary vascularity in a 39-year sickle cell disease patient. Right heart catheterization confirmed pulmonary hypertension with mPAP of 30 mmHg, pulmonary capillary wedge pressure of 10 mmHg, pulmonary vascular resistance of 4 woods, and left ventricular end-diastolic pressure of 8 mmHg.

Definitive diagnosis requires RHC. The brain natriuretic peptide (BNP) level is an indirect measure of PH. Thus, BNP levels of ≥160 pg/mL have a positive predictive value of 78%.^47^ Chest X-ray may show dilated main pulmonary artery and hypervascular markings (Figure 3).

Echocardiography is another non-invasive investigational tool.^48^ In one study, of 18 diagnosed on the basis of echocardiography criteria, 17 had PH as assessed with RHC.^44^

The management of PH in SCD includes general measures such as hydroxyurea, preventing hemolysis, and treating hypoxemia. Specific therapies such as phosphodiesterase 5 inhibitors, endothelin receptor antagonists, and prostacyclin analogs have been used, without significant improvement.

## Phosphodiesterase 5 inhibitors

In another study, sildenafil was used for around 6 months in 12 SCD patients with PH. It decreased the patients’ mean pulmonary artery pressure from 50 mm Hg to 41 mm Hg (*p*=0.04) and increased their mean 6-minute walk distance (6MWD) from 384 meters to 462 meters (*p*=0.0012).^49^ Another study was carried out in >74 SCD patients with high TRV, of whom 37 received sildenafil. However, the study was discontinued because of a high prevalence of serious adverse events in the sildenafil group. Sildenafil is thought to lower pain thresholds in patients with SCD.^50^

## Endothelin receptor blockade

In a cohort study, of 14 SCD patients with PH, 8 received ET receptor antagonists for >6 months. The 6MWD of the patients who received ET receptor improved from 357 meters to 398 meters (*p*=0.05). The BNP and TRV were decreased. Pulmonary artery pressures improved in 3 patients who underwent RHC. Adverse effects such as increased serum alanine aminotransferase (2 cases), worsening peripheral edema (4 cases), rash (one case), headache (3 cases), and decreased hemoglobin (2 cases) were observed. The therapy was discontinued in 2 patients.^51^

## Arginine

In patients with SCD, the arginine level is decreased because of increased arginase activity. A study in 10 SCD patients with PH showed that administration of oral arginine reduced the pulmonary artery systolic pressure by 15% after 5 days of treatment (*p*=0.002).^52^

## Prostacyclin

It has been used in patients with SCD associated with severe PH and right heart failure. Long-term prostacyclin infusion was administered in 2 patients but was discontinued in one patient because of its side effect profile; the other patient showed hemodynamic improvement.^46^

Further studies should evaluate the impacts of hydroxyurea, chronic blood transfusion, and anticoagulation on the disease course of PH in patients with SCD.

In conclusion, the acute and chronic pulmonary complications of SCD cause profound morbidity and mortality. Preventive measures must be taken into consideration, and early recognition of such complications and identification of their etiologies are crucial for the management of the disease. The initiation of empirical therapy with a low threshold for blood transfusion could prevent catastrophic deterioration. Screening for PH and SCLD should be established in patients with persistent respiratory symptoms and history of repeated ACS. More studies with large numbers of patients should be carried out for better understanding of these complications and to determine the effect of chronic blood transfusion in high-risk groups.
